# Elucidating the role of liver enzymes as markers and regulators in ovarian cancer: a synergistic approach using Mendelian randomization, single-cell analysis, and clinical evidence

**DOI:** 10.1186/s40246-024-00642-4

**Published:** 2024-06-24

**Authors:** Yinxing Zhu, Min Jiang, Zihan Gu, Hongyu Shang, Caiyin Tang, Ting Guo

**Affiliations:** 1 Taizhou Affiliated Hospital of Nanjing University of Chinese Medicine, Taizhou, 225300 China; 2 Department of Rehabilitation, The Affiliated Taizhou People’s Hospital of Nanjing Medical University, Taizhou, 225300 China; 3 Nanjing University of Finance & Economics, Nanjing, 210023 China; 4 Taizhou Polytechnic College, Taizhou, 225300 China; 5 Department of Imaging, The Affiliated Taizhou People’s Hospital of Nanjing Medical University, Taizhou, 225300 China; 6 Institute of Clinical Medicine, The Affiliated Taizhou People’s Hospital of Nanjing Medical University, Taizhou School of Clinical Medicine, Nanjing Medical University, Taizhou, 225300 China

**Keywords:** Ovarian cancer (OC), Alkaline phosphatase (ALP), Aspartate aminotransferase (AST), MR analysis, Single-cell analysis, Clinical data

## Abstract

**Objective:**

To investigate the association between liver enzymes and ovarian cancer (OC), and to validate their potential as biomarkers and their mechanisms in OC. Methods

Genome-wide association studies for OC and levels of enzymes such as Alkaline phosphatase (ALP), Aspartate aminotransferase (AST), Alanine aminotransferase, and gamma-glutamyltransferase were analyzed. Univariate and multivariate Mendelian randomization (MR), complemented by the Steiger test, identified enzymes with a potential causal relationship to OC. Single-cell transcriptomics from the GSE130000 dataset pinpointed pivotal cellular clusters, enabling further examination of enzyme-encoding gene expression. Transcription factors (TFs) governing these genes were predicted to construct TF-mRNA networks. Additionally, liver enzyme levels were retrospectively analyzed in healthy individuals and OC patients, alongside the evaluation of correlations with cancer antigen 125 (CA125) and Human Epididymis Protein 4 (HE4).

**Results:**

A total of 283 single nucleotide polymorphisms (SNPs) and 209 SNPs related to ALP and AST, respectively. Using the inverse-variance weighted method, univariate MR (UVMR) analysis revealed that ALP (*P* = 0.050, OR = 0.938) and AST (*P* = 0.017, OR = 0.906) were inversely associated with OC risk, suggesting their roles as protective factors. Multivariate MR (MVMR) confirmed the causal effect of ALP (*P* = 0.005, OR = 0.938) on OC without reverse causality. Key cellular clusters including T cells, ovarian cells, endothelial cells, macrophages, cancer-associated fibroblasts (CAFs), and epithelial cells were identified, with epithelial cells showing high expression of genes encoding AST and ALP. Notably, TFs such as TCE4 were implicated in the regulation of GOT2 and ALPL genes. OC patient samples exhibited decreased ALP levels in both blood and tumor tissues, with a negative correlation between ALP and CA125 levels observed.

**Conclusion:**

This study has established a causal link between AST and ALP with OC, identifying them as protective factors. The increased expression of the genes encoding these enzymes in epithelial cells provides a theoretical basis for developing novel disease markers and targeted therapies for OC.

**Supplementary Information:**

The online version contains supplementary material available at 10.1186/s40246-024-00642-4.

## Introduction

Ovarian cancer (OC) poses a significant global challenge in the field of gynecological malignancies due to its high recurrence and mortality rates, which seriously threaten women's health. Unfortunately, the lack of effective screening tools and challenges in early diagnosis contribute to the fact that 80% of OC patients are diagnosed at an advanced stage [1, 2]. Moreover, within two years after treatment, 50–70% of patients experience recurrence, resulting in a poor 5-year survival rate of 30% 3, 4. In current clinical practice, traditional tumor markers like Cancer Antigen 125 (CA125) and Human Epididymis Protein 4 (HE4) play a crucial role in aiding the diagnosis of ovarian cancer and evaluating the effectiveness of tumor treatment. However, despite devoted efforts in diagnosing and treating ovarian cancer over the past few decades, the persistent issue of a gloomy prognosis remains [3, 5]. Therefore, it is imperative to delve into the mechanisms underlying OC and explore novel therapeutic targets to enhance its management and improve patient outcomes.

Serum liver enzymes, including alanine aminotransferase (ALT), aspartate aminotransferase (AST), gamma-glutamyl transferase (GGT), and alkaline phosphatase (ALP), are widely recognized as the primary biomarkers for assessing liver injury. Observational studies have reported associations between these liver enzymes and various intrahepatic and extrahepatic diseases [6, 7]. In addition, elevated concentrations of liver enzymes in the blood have been suggested as an early indicator of increased risk for the four most common cancers: breast, prostate, colorectal, and lung [8]. However, whether there are causal relationships between liver enzymes and OC require further clarification.

Mendelian randomization (MR) analysis is considered a promising epidemiological method for accurately assessing potential causal relationships between exposure factors and outcomes. MR is likened to a randomized controlled trial, where the random allocation of allele genes assists in the random assignment of exposure. Moreover, MR methods are independent of environmental risk factors and operate prior to disease progression [9]. Therefore, to avoid reverse causation and potential confounding factors, genetic variations are employed as instrumental variables (IV) in MR analysis [10]. Utilizing summary statistics from past Genome-wide Association Studies (GWAS), MR analysis enables a more feasible exploration of potential causal relationships between exposure factors and ovarian cancer.

**Fig. 1:**
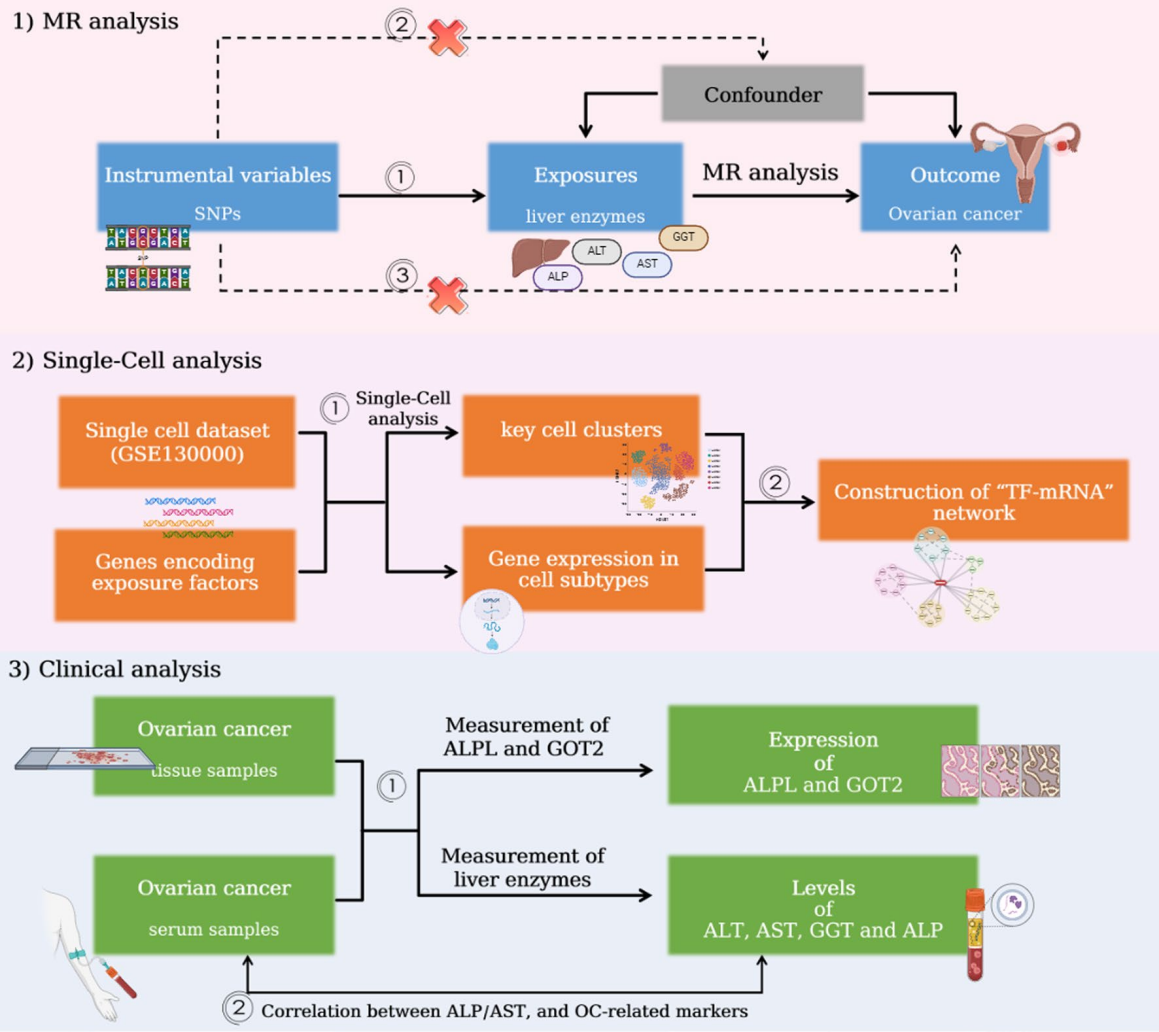
The schematic representation of this study

To further explore the potential etiology, novel markers and pathogenic mechanisms of OC, in this study, MR analysis was employed to identify enzymes with a significant causal relationship with OC. Furthermore, the expression of genes encoding exposure-related factors in single cells was analyzed, the regulatory mechanisms underlying OC were investigated, and the clinical data of OC patients were identified, providing significant insights into the pathogenesis of OC. From a genetic perspective, the study observes and confirms the causal mechanisms between these factors, providing more reliable evidence for causal inference (Fig. 1).

## Materials and methods

### Data source

The outcome and exposure factors datasets were obtained from Integrative Epidemiology Unit Open genome-wide association statistics (IEU OpenGWAS) database (https://gwas.mrcieu.ac.uk/) [11]. The ovarian cancer (OC) dateset (ieu-a-1120), as outcome, contained 25,509 cases and 40,941 normal samples with European people, a variety of enzymes as exposure factors, including the alkaline phosphatase (ALP, ukb-d-30610_irnt contained 13,586,006 single nucleotide polymorphisms (SNPs), aspartate aminotransferase (AST, ukb-d-30650_irnt included 13,586,009 SNPs), alanine aminotransferase (ALT, ukb-d-30620_irnt contained 13,586,000 SNPs), and gamma-glutamyltranspeptidase (GGT, prot-a-1208 covered 10,534,735 SNPs). GSE130000, as single-cell dateset, was compiled from Gene Expression Omnibus (GEO; https://www.ncbi. nlm.nih.gov/geo/). Then 4 primary tumors, 2 peritoneal metastases and 2 relapse tumors samples were selected from GSE130000 for single-cell analysis.

We collected patients with ovarian cancer (diagnosed by pathology) who were admitted at the gynecologic Department, Taizhou People's Hospital affiliated to Nanjing Medical University from January 2017 to December 2022. The clinical data of the corresponding patients were extracted from the patient files. The physical examination data of patients with OC 2 years before the initial diagnosis were collected through the electronic medical record system, physical examination system and the way provided by the patients themselves. In addition, healthy female volunteers who underwent a routine physical examination yearly were as healthy controls. All individuals aged at 25–65 years and met the FIGO stage of I/II. Patients who have received chemo or radiotherapy before surgery were excluded from this study. Patients with alcohol consumption, liver disease (such as viral hepatitis, immune liver disease, etc.), family history of liver disease and incomplete data were excluded. Ultimately, 130 pre-ovarian cancer (pre-OC) patients and 150 healthy controls were included in the study. Among the 130 pre-OC patients, the histological subtypes confirmed by subsequent surgery were: 102 were serous ovarian cancer, 14 were endometrioid ovarian cancer, 5 were mixed ovarian cancer, and 9 were clear cell ovarian cancer. The Ethics Committee approved this project of The Affiliated Taizhou People's Hospital of Nanjing Medical University following the Declaration of Helsinki (approval number, KYKY 2019077).

### Selection of instrumental variable (IVs)

To ensure valid IVs values, three basic assumptions of MR analysis should be satisfied: (1) IVs are closely related to exposure factors, (2) IVs cannot be associated with any confounding factors, (3) Exposure is the only way that genetic variation can affect the outcome [12]. Moreover, these genome-wide relevance SNPs (*p* < 5 × 10^–8^) were used as IVs. They were tested for independent inheritance (r^2^ = 0.001, kb = 10,000) without linkage disequilibrium (LD) among themselves. This study also estimated r^2^ for the exposure variance interpreted by each IV [13].

### Mendelian randomization (MR) analysis of multiple enzymes and OC

The causal effects of multiple enzymes and OC were explored through univariate MR analysis (UVMR) using TwoSampleMR package (v0.5.6) [14]. Then five algorithms were applied to MR analysis, including MR-Egger regression, inverse-variance weighted (IVW) method, the weighted median test, the weighted mode test and the simple mode test [15]. The IVW test was primary method for studying the causal relationship between OC and multiple enzymes. If the assumption that all included SNPs can be used as valid IVs was satisfied, the IVW method provided an accurate estimate 16. The other methods were used as supplementary analysis methods. Further, sensitivity analysis was utilized to assess the reliability of MR results, a heterogeneity test was performed by MR Egger and IVW methods, and the results were quantified by the Cochrane Q test. If Q-*p* value > 0.05, it indicated that there was no heterogeneity between two datasets. In order to evaluate the potential multidirectional effects of IVs, MR-Egger regression was adopted. If the *p* value > 0.05, it suggested that there were no confounding factors and no potential pleiotropy in this study. The Leave-One-Out (LOO) method was used to see if there were outliers in the effect of each IVs. Enzymes with *p* values less than 0.05 in the IVW method were considered as exposure factors for OC and multivariate MR analysis (MVMR) was performed in a manner consistent with UVMR. Eventually, steriger test was performed to rule out reverse causal effects.

### Analysis of enzyme-related genes causally associated with OC at the single-cell level

In order to analyze the expression of genes associated with enzymes that have a causal relationship with OC at the single-cell level, we used GSE130000 for single-cell analysis. The Seurat package (v4.0.5) [17] was utilized to quality control and filter the data of GSE130000. The genes with expression data in at least three cells and the number of genes detected in more than 100 cells were selected as screening conditions, and NormalizeData was applied to standardize the data. Vst was used to select 2000 hypervariable genes for subsequent analysis. This data was dimensionally reduced by principal component analysis (PCA). Afterward, the FindNeighbors and FindClusters functions performed unsupervised clustering analysis of the cells and the clustering was visualized using umap. Meanwhile, genes corresponding to exposure factors were found as genes encoding exposure factors using GeneCards (https://www.genecards.org/), and gene expression in cell subtypes was explored. In addition, functional enrichment analysis of different cell types was also performed using ReactomeGSA (v1.4.2), and CellPhoneDB was applied to analyze intercellular ligand-receptor interactions.

In order to observe the differentiation trajectory of cells, the FindNeighbors and FindClusters functions perform unsupervised cluster analysis on the cells, and umap was also used to visualize the clusters. GSVA (v1.38.2) was applied to calculate the HALLMARK pathway score for each cell, then the differentialGeneTest function was utilized to identify genes that differed between different subtypes of cells, and finally monocle (v2.18.0) package [18] was used for quasi-time series analysis.

### Construction of “TF-mRNA” network

The NetworkAnalyst (https://www.networkanalyst.ca/) database was applied to predict transcription factors (TFs) targeting genes encoding exposure factors, and TF-mRNA networks were constructed. In addition, highly expressed TFs were extracted from the single-cell dataset and subnetworks were constructed.

### Statistical analysis

MR and Single-Cell analyses were performed in R (version 4.2.3) software. SPSS 23.0 software (SPSS, Chicago, IL) was utilized to analyze the clinical data. The study results were presented as means ± standard deviation (SD). Shapiro–Wilk test was used to determine whether the sample data fit the normal distribution.Student’s t-test were used for normal distribution, and Mann–Whitney test was used for non-normal distribution. Correlation between the expression and the clinical characteristic of the OC patients was analyzed by chi-square test. The correlations between liver enzymes and OC related biomarkers were analyzed by linear regression analysis. *P*-value of less than 0.05 indicated statistical significance.

## Results

### Identification of causal relationship of ALP and AST on OC via UVMR analysis

**Fig. 2:**
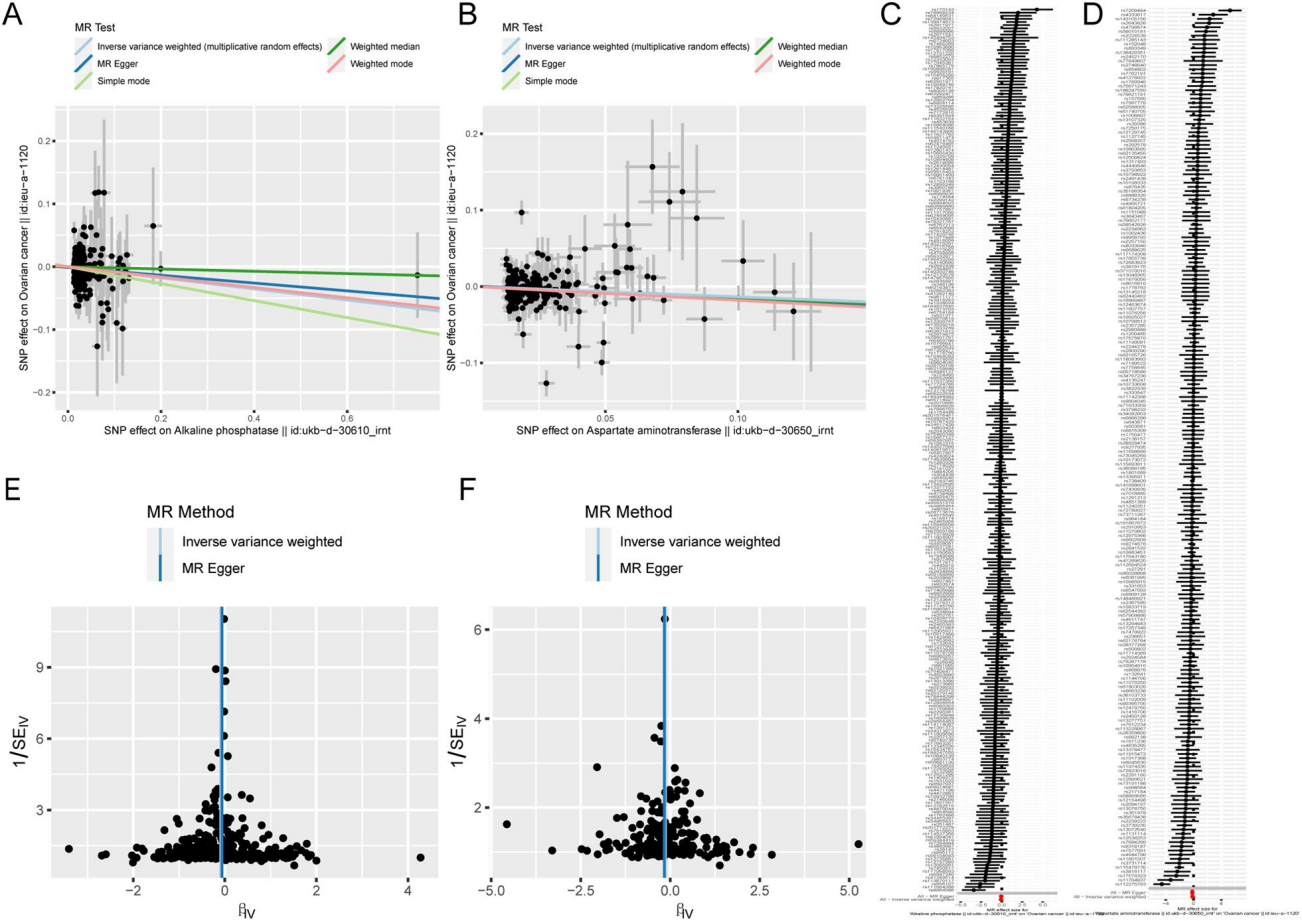
The UVMR analysis results. **A**, **B** The scatter plots of ALP and AST. **C**, **D** The forest plots of ALP and AST. **E**, **F** The funnel plots of ALP and AST

A total of 283, 209 and 187 SNPs were obtained that related to ALP, AST and ALT, respectively. Regrettably, no SNP associated with GGT was identified, precluding further investigation into GGT in this study. Based on IVW results, the relationship between ukb-d-30610_irnt and ieu-a-1120 (*p* = 0.005, Odd ratio [OR] = 0.938), between ukb-d-30650_irnt and ieu-a-1120 (*p* = 0.017, OR = 0.906) all satisfied *p* < 0.05, on the contrary, the relation between ukb-d-30620_irnt and ieu-a-1120 pleased *p* > 0.05 (Table 1), suggesting that only ALP and AST had notable causal relationship with OC respectively, and OR < 1, indicating that ALP and AST were protective factors for OC. Meanwhile, the scatter plot showed that the slope of IVW was negative and there was no intercept (Fig. 2A, B), and the forest plot showed that the overall effect of IVW method was less than 0 (Fig. 2C, D), which comprehensively indicated that ALP was a protective factor for OC. In addition, SNPs were randomly and uniformly distributed on both sides of the IVW line, indicating that MR conforms to Mendelian's second law (Fig. 2E, F). Further, to verify the reliability and robustness of MR analysis, *p-*value of Cochran Q test was less than 0.05, because of the result of IVW was less than 0.05, manifested that heterogeneity between the two data sets were not evident effects on the results (Table 2). Horizontal pleiotropy illustrated the absence of potential confounders (Table 2). The LOO analysis provided further evidence that causality was not driven by any single SNP (Fig. S1A, B). Low or no heterogeneity, no sign of potentially confounding SNPs, and similar results from robust analyses of pleiotropy all implicitly guarantee that MR assumptions (2) and (3) hold.

**Table 1:** The results of UVMR analysis

Exposure	Method	nSNP	b	se	*p* value	OR
ALP	IVW	283	− 0.088	0.031	0.005	0.938
AST	IVW	209	− 0.135	0.057	0.017	0.906
ALT	IVW	187	− 0.064	0.049	0.192	0.954

**Table 2:** Heterogeneity and pleiotropy analyses of ALP and AST on OC

Exposure	Heterogeneity						Pleiotropy test		
	MR egger			IVW					
	Q	Q df	Q *p* value	Q	Q df	Q *p* value	Egger intercept	SE	*p* value
ALP	336.428	281	0.013	337.019	282	0.014	− 0.001	0.002	0.483
AST	331.411	207	< 0.001	331.542	208	< 0.001	0.001	0.003	0.775

### Discussion of causal relation of ALP and AST with OC through MVMR analysis

**Fig. 3:**
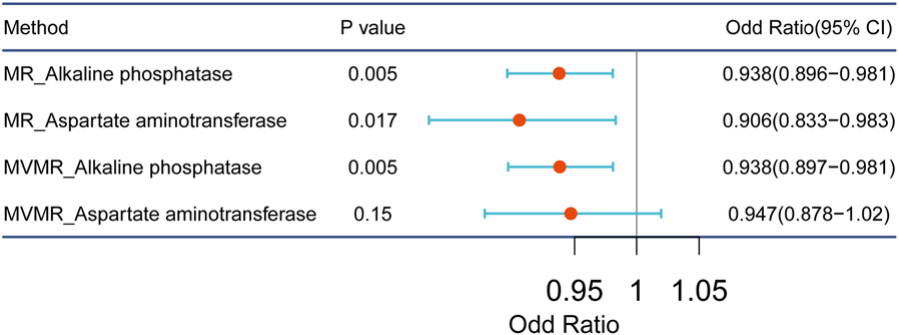
Forest plot of the results of UVMR and MVMR analysis

A total of 337 SNPs as IVs were strongly related to ALP and AST. The IVW results showed that the relationship between ukb-d-30610_irnt and ieu-a-1120 (*p* = 0.005, OR = 0.938) satisfied *p* < 0.05, while the relation between ukb-d-30650_irnt and ieu-a-1120 (*p* = 0.150, OR = 0.947) satisfied *p* > 0.05 (Table 3), suggesting that ALP was still a significant protective factor for OC, while ASP became insignificant after correction by ALP (Fig. 3). In other words, ALP had a more direct effect on OC. In addition, they passed the steriger test (Table 3), which further confirmed the reliability of MR results.

**Table 3:** The results of MVMR analysis

Exposure	nSNP	b	se	*p* value	OR	correct_causal_direction	Steiger *p* value
ALP	214	− 0.088	0.031	0.005	0.938	TRUE	0
AST	142	− 0.075	0.052	0.150	0.947	TRUE	5.41^E−302^

### Single cell data analysis

**Fig. 4:**
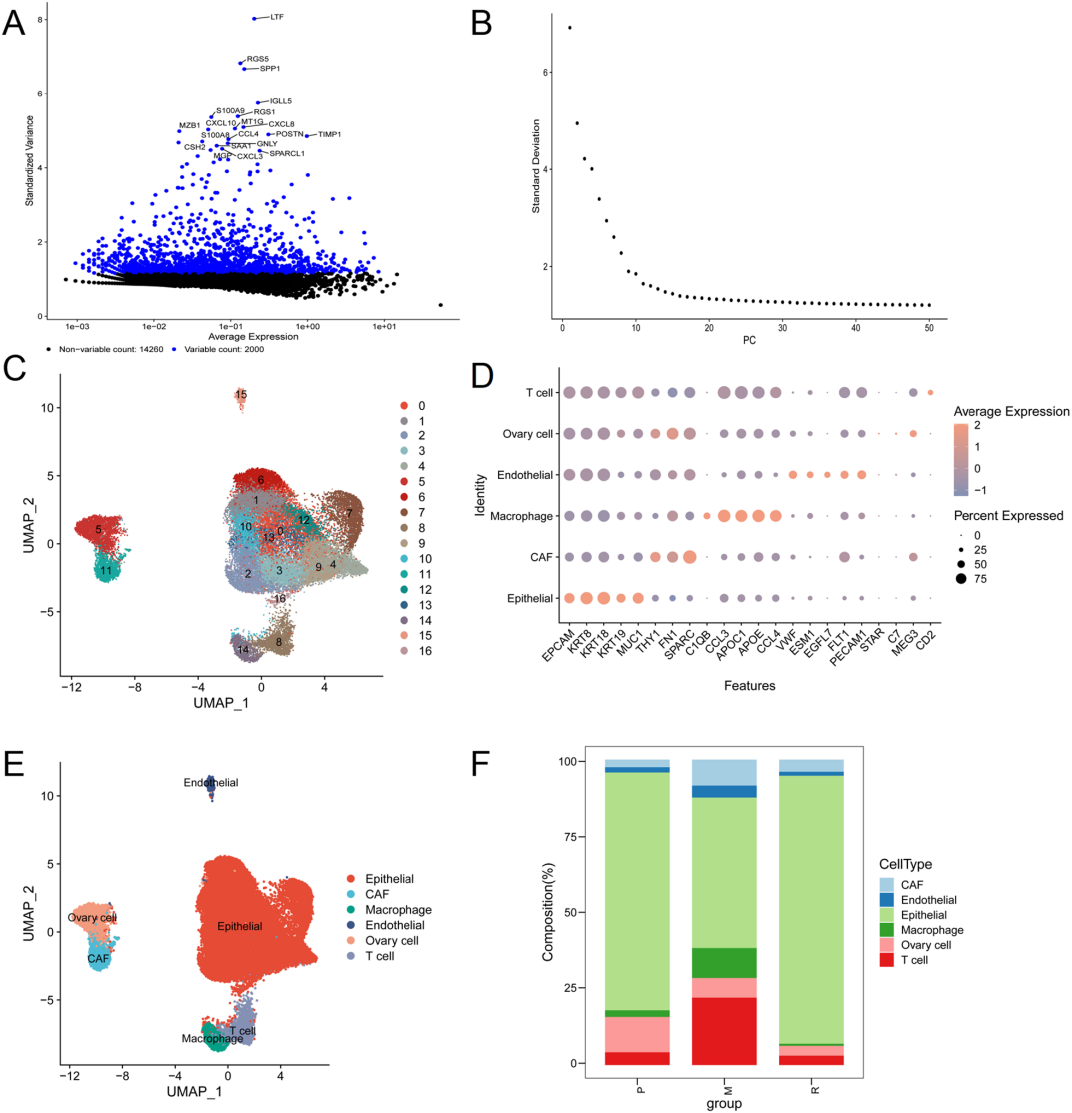
Single cell data analysis. **A** Intercellular expression of highly variable genes. **B** Examine and visualize PCA results with ElbowPlot. **C** Visualization of cell clusters. **D** Dot plot of marker gene expression for each cluster. **E** Umap cell cluster distribution map of cell subsets. **F** Histogram of cell proportion in each group (P represents primary, M represents metastasis, and R represents recurrence)

In all 2000 highly variable genes were selected for analysis (Sample GSM3729170_P1 was used for display) (Fig. 4A, Fig. S2), and the PC in the first 30 inflection points were selected for cluster analysis by PCA dimensionality reduction (Fig. 4B). By unbiased clustering based on UMAP, 17 cell clusters were identified (Fig. 4C). A total of six cell subtypes were annotated (Fig. 4D) (T cell, Ovary cell, Endothelial, Macrophage, CAF and Epithelial). Interestingly, the proportion of Epithelial was the highest in the three groups (primary tumor, peritoneal metastasis and relapse tumors) (Fig. 4E).

### Main expression of the genes encoding exposure factors in epithelial cells

**Fig. 5:**
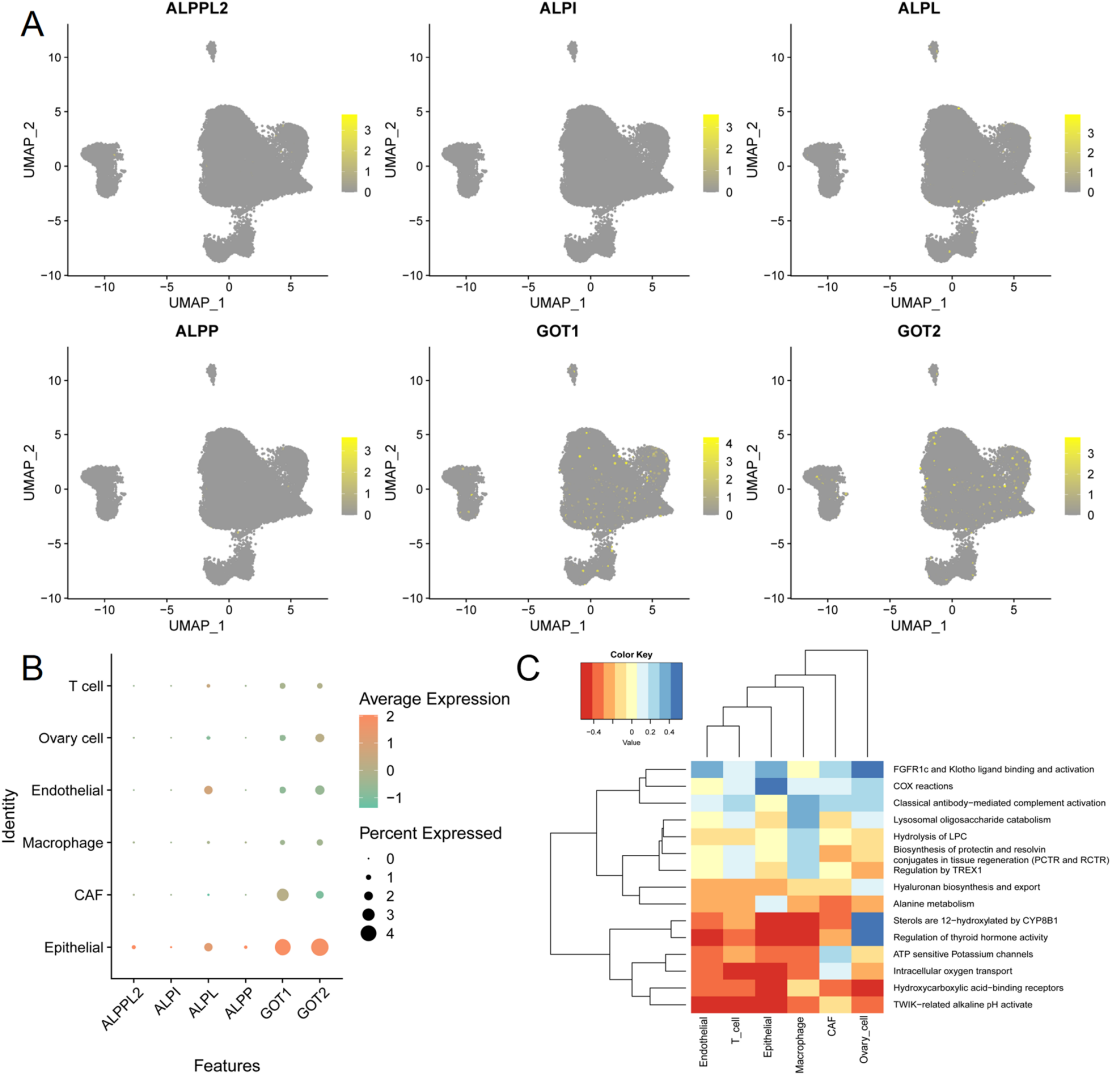
Main expression of the genes encoding exposure factors in epithelial cells. **A** Umap visualization of genes encoding exposure factors. **B** Dot plots of gene expression encoding exposure factors. **C**. Reactome pathway between cells

Furthermore, the corresponding genes encoding ALP and AST were identified using the GeneCards database (https://www.genecards.org/). AST was encoded by *GOT2, GOT1* and *GOT1L1*. ALP was encoded by *ALPL*, *ALPP*, *ALPI*, and *ALPG* (Table 4). Genes encoding exposure factors were looked up using GeneCards and found to be more expressed in the Epithelial (Fig. 5A, B). Furthermore, Epithelial was enriched in numerous pathways, such as Hydroxycarboxylic acid-binding receptors, Intracellular oxygen transport, TWIK-related alkaline pH activate, Sterols are 12-hydroxylated by *CYP8B1* and Regulation of thyroid hormone activity (Fig. 5C).

**Table 4:** The genes encoding exposure factors

Exposure	Gene	Description	Category
ALP	ALPL	Alkaline Phosphatase, Biomineralization Associated	Protein coding
	ALPP	Alkaline Phosphatase, Placental	Protein coding
	ALPI	Alkaline Phosphatase, Intestinal	Protein coding
	ALPG	Alkaline Phosphatase, Germ Cell	Protein coding
AST	GOT2	Glutamic-Oxaloacetic Transaminase 2	Protein coding
	GOT1	Glutamic-Oxaloacetic Transaminase 1	Protein coding
	GOT1L1	Glutamic-Oxaloacetic Transaminase 1 Like 1	Protein coding

### Cell communication analysis

**Fig. 6:**
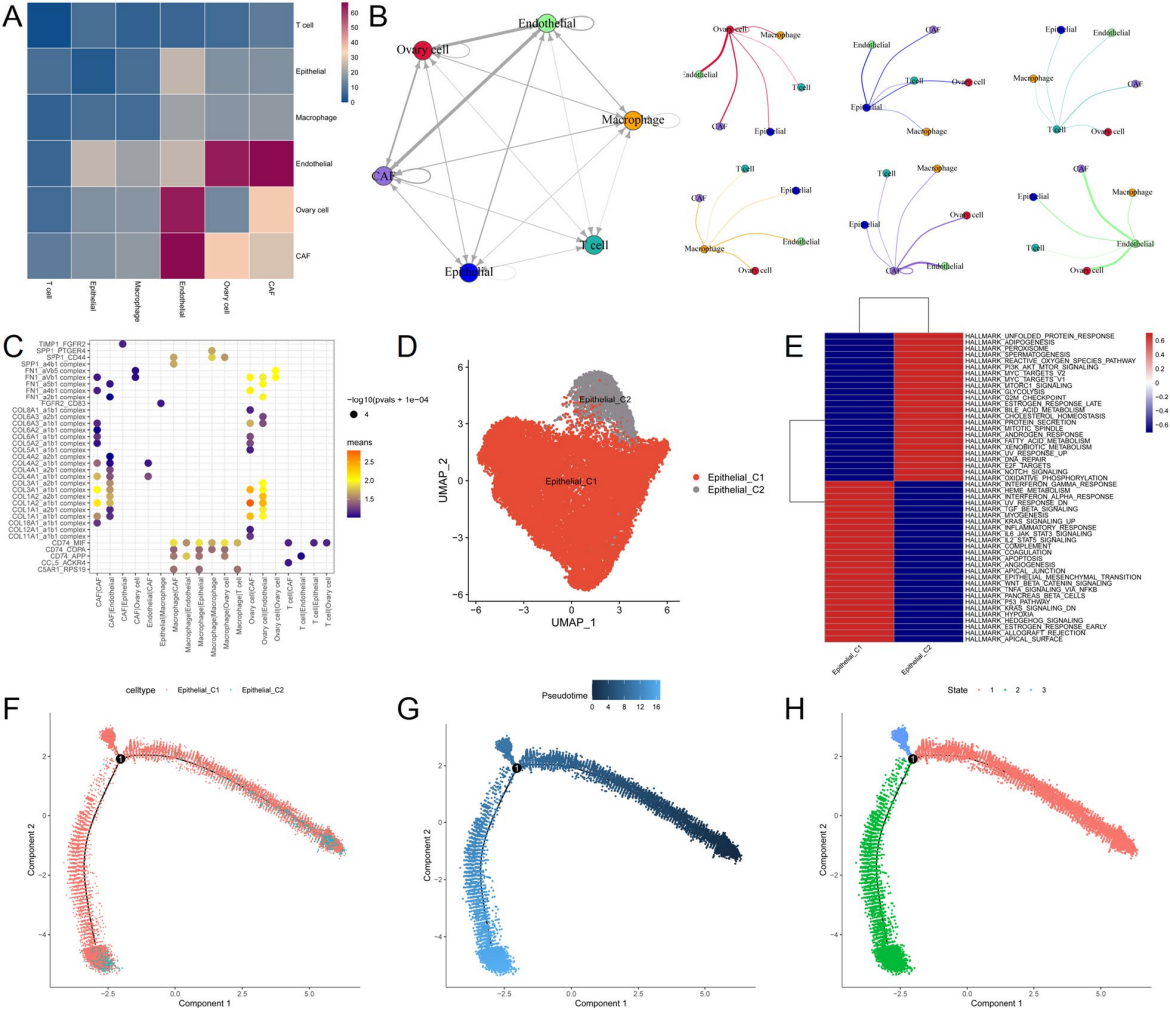
Cell communication analysis. **A** Heat map of the number of interacting ligand-receptors between each cell subpopulation. **B** Interaction networks between cell subsets. **C** Visualization of cell–cell receptor ligands. **D** A umap visualization of epithelial cell clustering. **E** Epithelial subclasses HALLMARK pathway. **F**–**H** Quasi-temporal analysis of epithelial cells

By CellPhoneDB analysis, it was found that endothelial cells had the largest number of receptor-ligands with epithelial cells, with 25 receptor-ligands (Fig. 6A, B). Importantly, TIMP1_FGFR2 between CAF and Epithelial, FGFR2_CD83 between Epithelial and Macrophage, C5AR1_RPS19, CD74_APP, CD74_COPA, CD74_MIF between Macrophage and Epithelial cell, and CD74_MIF between T cell and Epithelial were a receptor ligand associated with Epithelial with *p* < 0.05 (Fig. 6C). Moreover, Epithelial cells were divided into two subtypes, Epithelial_C1 and Epithelial_C2 (Fig. 6D). The HALLMARK pathway of the subtype was shown in the Fig. 6E. In order to explore the differential genes in the different components of epithelial cells, the results showed that epithelial cells existed in three different differentiation states. It was found from the State2 branch that C2 had a higher degree of differentiation than C1 (Fig. 6F–H).

### Establishment of “TFs-mRNAs” network

**Fig. 7:**
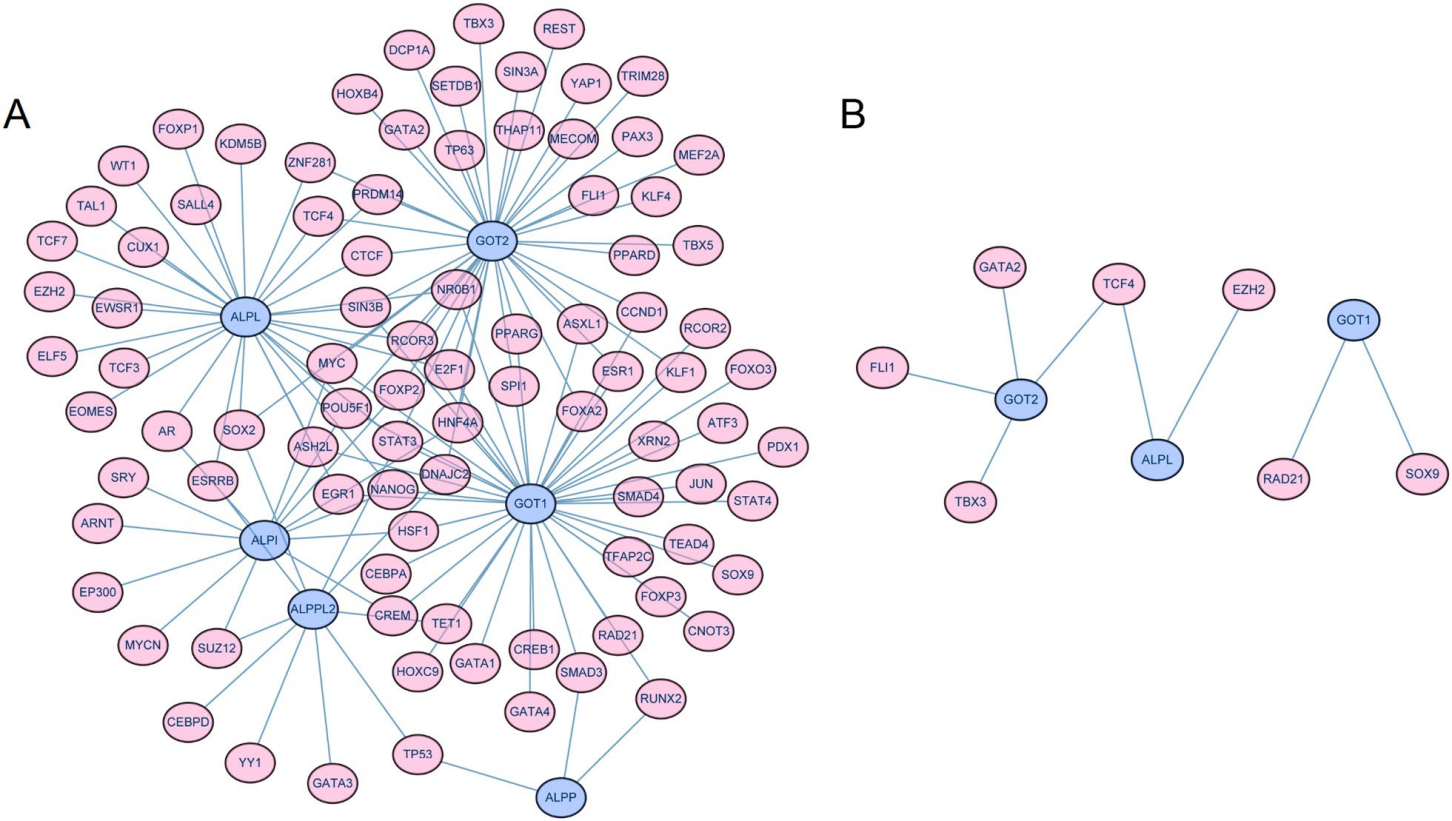
“TFs-mRNAs” network. **A** TFs-mRNAs network of genes encoding exposure factors. **B** Subnetwork of TFs-mRNAs network

A total of 90 TFs were acquired and a network of TF-mRNAs containing 96 nodes with 137 edges was constructed, with some relationships like *GOT2* and THAP11, *ALPPL2* and SOX2 in the network (Fig. 7A). In addition, highly expressed TFs in single-cell data were extracted, such as TCF4 and EZH2, where TCF4 had relationships with both *GOT2* and *ALPL* (Fig. 7B).

### Identification of the expressions of liver enzymes in pre-OC and OC patients

**Fig. 8:**
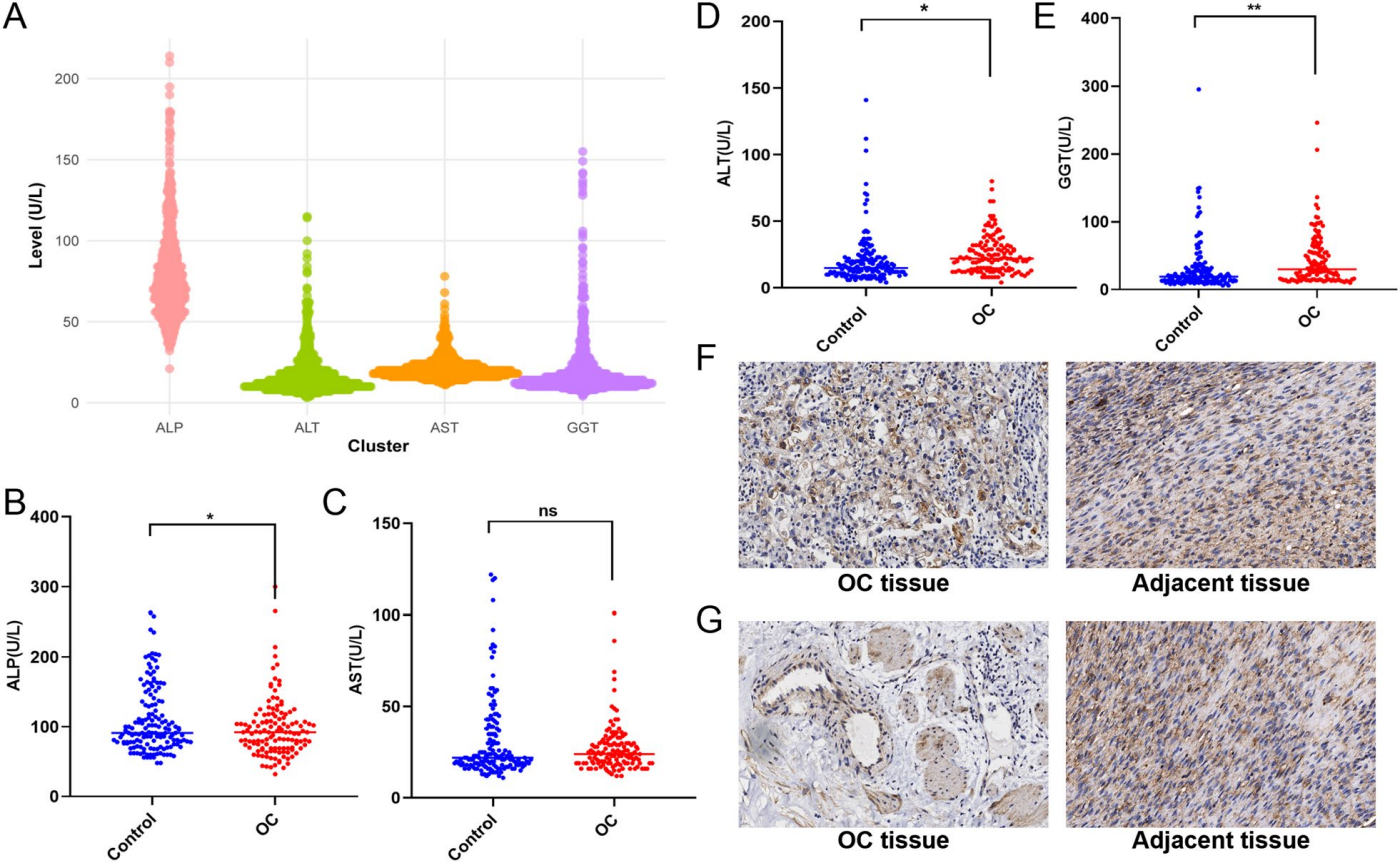
Clinical data analysis. **A** Distribution of liver enzyme levels. **B–D** The level of liver enzymes between OC patients and healthy controls (*p < 0.05, **p < 0.01). **F** The expressions of ALPL in tissues. **G** The expressions of GOT2 in tissues

Data on hepatic enzyme levels were extracted from health examination records of 71,682 female patients who were screened at the Taizhou People's Hospital affiliated to Nanjing Medical University from January 2015 to December 2022. All individuals aged at 25–65 years. The median values for ALP, AST, ALT, and GGT were determined to be 70 U/L, 19 U/L, 14 U/L, and 15 U/L, respectively. Furthermore, a subset of 1,143 female patients was randomly selected for the visualization of the distribution of liver enzyme levels Fig. 8A.

Consequently, we operationally defined elevated liver enzyme levels as values greater than the median, and levels lower or equal to the median as indicative of low liver enzyme status. A comparative analysis was conducted on the liver enzyme levels of 130 pre-ovarian cancer (pre-OC) patients and 150 healthy controls. It was observed in Table 5 that females with elevated levels of ALP had a relatively lower risk of developing OC *p* = 0.036). Conversely, elevated levels of ALT and GGT were associated with a higher risk of OC (*p* = 0.001 and *p* = 0.047, respectively). Meanwhile, as shown in Fig. 8B–E, ALP was decreased in serum of pre-OC patients compared with healthy control (*p* < 0.05). Interestingly, ALT (*p* < 0.05) and GGT (*p* < 0.01) were increased in serum of pre-OC patients compared with healthy control. Moreover, expressions of ALPL and GOT2 were found to be down-regulated in OC tissues compared with the cancer adjacent tissues Fig. 8F, G.

**Table 5:** Incidence of ovarian cancer with different exposure value

	Healthy	OC	Ratio (%)	*p* value	OR
ALP↑	122	93	43.26	0.036	0.99
ALP-	28	37	56.92		
AST↑	92	93	50.27	0.206	0.99
AST-	58	37	38.95		
ALT↑	97	91	48.40	0.001	1.02
ALT-	53	39	42.39		
GGT↑	76	91	54.49	0.047	1.01
GGT-	74	39	34.51		

### Correlation between liver enzymes and OC-related markers

**Fig. 9:**
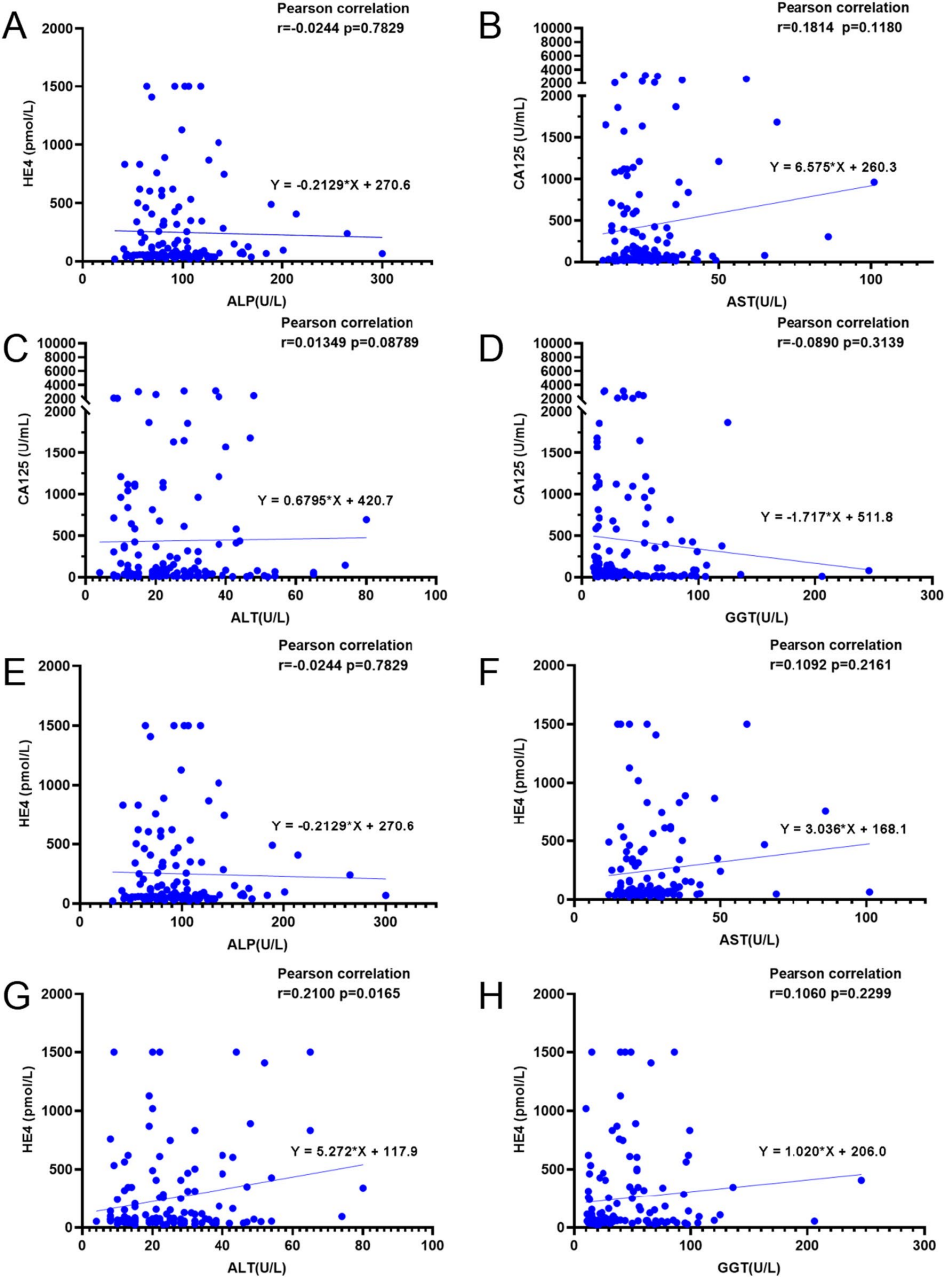
Correlation between liver enzymes and OC-related markers. **A**–**D** Correlation between ALP/AST/ALT/GGT and CA125, respectively; **E**–**H**. Correlation between ALP/AST/ALT/GGT and HE4, respectively

As shown in Fig. 9, the level of ALP was negatively correlated with CA125 (r = − 0.2364, *p* = 0.0068); on the other hand, the level of ALP indicated no correlated with HE4 activity (r = − 0.0244,* p* = 0.7829); and AST was also no correlated with CA125 (r = 0.1814, *p* = 0.1180) and HE4 (r = 0.1092, *p* = 0.2161). Besides, the level of ALT was positively correlated with HE4 (r = 0.2100, *p* = 0.0165); on the other hand, the level of ALP indicated no correlated with CA125 activity (r = 0.01349, *p* = 0.08789); and GGT was also no correlated with CA125 (r = 0.0890, *p* = 0.3139) and HE4 (r = 0.1060, *p* = 0.2299).

## Discussion

Ovarian cancer (OC), a highly lethal malignancy, significantly impacts the female reproductive system and is associated with high mortality rates globally. It is frequently diagnosed in advanced stages due to the general absence of early symptoms and the lack of effective screening strategies [3, 19]. Liver enzymes are routine biomarkers in physical examinations and are associated with a variety of diseases. They serve as potential biomarkers for the early diagnosis of OC. Consequently, this study employed Mendelian randomization analysis, which is a statistical method that uses genetic variants as instrumental variables (commonly SNPs) to infer causal relationships between an exposure and an outcome, to identify enzymes with a significant causal relationship to OC. SNPs can influence gene expression through multiple mechanisms such as altering protein sequence/function via coding region SNPs, modulating transcriptional activity by regulatory region SNPs, and affecting RNA splicing patterns from splice site SNPs, and so on [20, 21].

Further analysis was conducted at the single-cell level to examine the expression of genes encoding exposure factors within cells, providing a preliminary investigation into the regulatory mechanisms affecting OC. Additionally, retrospective clinical data were utilized for validation purposes.

The MR analysis results from this study revealed a causal relationship between ALP and AST with OC, and both were identified as protective factors. As a univariate factor, AST was significant, but in the multivariate analysis, its effect was corrected by ALP, transitioning from a significant risk factor to a non-significant factor. In other words, this suggests that the impact of ALP as a risk factor for OC is more direct compared to the influence of AST. While some degree of heterogeneity (*p*-value < 0.05) existed in the MR analysis, the IVW method used in this study requires the SNPs to fully meet the three principles of MR research in order to obtain a correct causal estimate. Therefore, the use of this method has not had a significant impact on the MR results. In the future, we will also conduct more analyses from various perspectives, such as subgroup analysis, to identify potential confounding factors, and adopt appropriate corrective measures to ensure the validity and accuracy of the final experimental conclusions.

ALP is a zinc-dependent dimeric metalloenzyme. Research into the enzymatic characteristics of ALP has elucidated that the active site encompasses a serine residue, with a proximate amino acid sequence reminiscent of serine environments [22]. In healthy adults, serum ALP levels are conventionally found to span from 40 to 150 U/L; deviations from this range are frequently indicative of pathological conditions [23]. Elevated serum ALP levels, for instance, have been correlated with Multiple Myeloma and osteoblastic bone tumors [24, 25]. The human body synthesizes a variety of amino transferases, with AST being predominantly localized in the mitochondrial cytoplasm of hepatocytes. Clinically, elevated serum AST levels can signify several conditions, encompassing viral hepatitis, alcoholic liver disease, cirrhosis, cholestatic syndrome, acute myocardial infarction, or skeletal muscle damage [26]. Recent investigations have uncovered a correlation between ALT/AST ratios and insulin resistance, particularly in early to middle-aged women [27]. Nevertheless, the potential causal link between the two liver enzymes’ elevation and OC remains to be definitively elucidated.

In our study, females with elevated levels of ALP had a relatively lower risk of developing OC. Conversely, elevated levels of ALT and GGT were associated with a higher risk of OC. And the clinical data have also substantiated discernible differences in ALP levels in patients up to two years prior to an OC diagnosis. The ALP levels measured two years before the diagnosis in OC patients were found to be lower than those in the healthy population during a comparable timeframe. Interestingly, ALT and GGT showed higher levels in pre-OC patients than those in the healthy population. Moreover, an inverse correlation was observed between the levels of ALP and the ovarian cancer biomarker CA125. In several cancer types, including colorectal cancer (CRC), breast cancer, and non-small cell lung cancer (NSCLC), low levels of alkaline phosphatase (ALP) have been associated with a worse prognosis for patients [28]. Some studies have observed a relationship between low ALPL expression and chemo-resistance of high-grade serous ovarian cancer (HGSOC) cells to paclitaxel. Low expression of ALPL was found to be inversely related to the FIGO stages and histological grades in a cohort of 90 patients with serous ovarian cancer (SOC). Moreover, they demonstrated that ALPL overexpression might decrease migration and invasion of HGSOC cells by inhibiting the WNT5A-FZD2-STAT3 signaling axis [29]. AST is encoded by GOT2. GOT2 can assume a tumor-suppressive role in certain oncogenic contexts, particularly in hepatocellular carcinoma. However, the precise mechanisms by which GOT2 exerts its effects in OC remain unclear at this time.

The single-cell data analysis reveals the cellular composition of ovarian tissue, including macrophages, ovarian cells, endothelial cells, epithelial cells, tumor fibroblasts, T cells, and others. AST is encoded by genes such as GOT1, GOT2, while ALP is encoded by ALPL, ALPP, ALPI, ALPG, with these genes primarily expressed in epithelial cells. Furthermore, genes associated with TWIK-related alkaline pH activate, Hydroxycarboxylic acid-binding receptor, Intracellular oxygen transport, Regulation of thyroid hormone activity, Sterols are 12-hydroxylated by CYP8B1 are enriched in epithelial cells.

The cell communication results indicate that endothelial cells have the most interactions with epithelial cells. Additionally, receptors and ligands associated with epithelial cells and having a *p* < 0.05 include TIMP1_FGFR2 between CAF (Cancer-Associated Fibroblast) and Epithelial cells, FGFR2_CD83 between Epithelial and Macrophage cells, C5AR1_RPS19, CD74_APP, CD74_COPA, and CD74_MIF between Macrophage and Epithelial cells, and CD74_MIF between T cells and Epithelial cells. Furthermore, clustering analysis of epithelial cells (divided into two clusters) and pseudotime analysis reveal three distinct differentiation states in epithelial cells, with Cluster 2 (C2) showing a higher degree of differentiation compared to Cluster 1 (C1). Subsequently, transcription factors encoding the exposure factors' genes were predicted using online databases. Finally, our pathological examinations of tissue samples revealed that the expression of ALPL and GOT2 in cancerous tissues was lower than that in adjacent non-tumorous tissues.

Recent literature extends the significance of ALP to oncological contexts. Rao et al. demonstrated that an ALPL knockdown could attenuate migration in prostate cancer cell lines [30]. Concurrently, another investigation posited that ALPL specifically mitigates lung adenocarcinoma (LUAD) cell metastasis by interacting with the *p*-ERK/c-Myc/RhoA signaling axis [31]. Moreover, overexpression of ALPL was observed to curtail migration and invasion in high-grade serous ovarian cancer (HGSOC) cell models [29].

GOT2 has been implicated in the augmentation of tumorigenicity. A recent study suggests that cutaneous melanoma (CM) patients with heightened GOT2 expression exhibit diminished survival rates and reduced immune cell infiltration [32]. Concurrently, elevated GOT2 levels have been linked to the accelerated proliferation of breast cancer cells [33]. Paradoxically, GOT2 can also assume a tumor-suppressive role in certain oncogenic contexts. For instance, GOT2 expression is inversely regulated in hepatocellular carcinoma (HCC) tissues, where its downregulation is associated with adverse prognostic outcomes in HCC patients. Functionally, GOT2 silencing has been shown to enhance proliferation, migration, and invasion of HCC cell lines [34]. While the underlying mechanisms of GOT2's dualistic role remain under active investigation, current evidence points to its involvement in the reprogramming of glutamine metabolism, a process that ostensibly supports cancer progression.

Given these findings, the emerging evidence concerning the role of ALPL and GOT2 in oncological pathophysiology, particularly with respect to their influence on OC, necessitates additional research. This is imperative to reconcile current discrepancies in the literature and to provide a deeper understanding of the molecular mechanisms at play.

However, it is noteworthy that a single biomarker often cannot fully account for a complex health condition, and typically, a comprehensive assessment requires the integration of multiple indicators and clinical information. This study was limited by a relatively small sample size and an observational period of only two years, which may introduce a certain degree of bias. In addition, the patients selected for clinical trials are of a wide age, and heterogeneity in this segment of the population may affect the study results. Further prospective studies should entail multi-center, long-term studies to expand the sample size and extend the duration of observation. Moreover, the underlying mechanisms by which ALP and GOT2 are involved in ovarian cancer (OC) pathogenesis warrant further investigation through in-depth cellular and animal studies.

In conclusion, our study leveraged MR analysis, single-cell data, and clinical data analysis to provide a theoretical basis for the mechanisms associated with ALP and AST in ovarian cancer.

### Supplementary Information

**Additional file 1.**

**Additional file 2.**

## Data Availability

The datasets generated during and/or analyzed during the current study are available from the corresponding author upon reasonable request.

## Abbreviations

OC: Ovarian cancer
AST: Aspartate aminotransferase
ALP: Alkaline phosphatase
GWAS: Genome-wide association studies
ALT: Alanine aminotransferase
GGT: Gamma-glutamyltransferase
IVW: Inverse-variance weighted
MR: Mendelian randomization
TF: Transcription factor
CA125: Cancer antigen 125
HE4: Human Epididymis Protein 4
SNP: Single nucleotide polymorphism
CAFs: Cancer-associated fibroblasts
IV: Instrumental variables
LD: Linkage disequilibrium
LOO: Leave-One-Out
PCA: Principal component analysis
CM: Cutaneous melanoma
LUAD: Lung adenocarcinoma
HGSOC: High-grade serous ovarian cancer
HCC: Hepatocellular carcinoma
OR: Odd ratio
