# Supplementary figures and images for: Elucidating the role of liver enzymes as markers and regulators in ovarian cancer: a synergistic approach using Mendelian randomization, single-cell analysis, and clinical evidence
Source: Hum Genomics. 2024 Jun 24;18:71. doi: 10.1186/s40246-024-00642-4 (PMC11197171; doi:10.1186/s40246-024-00642-4)

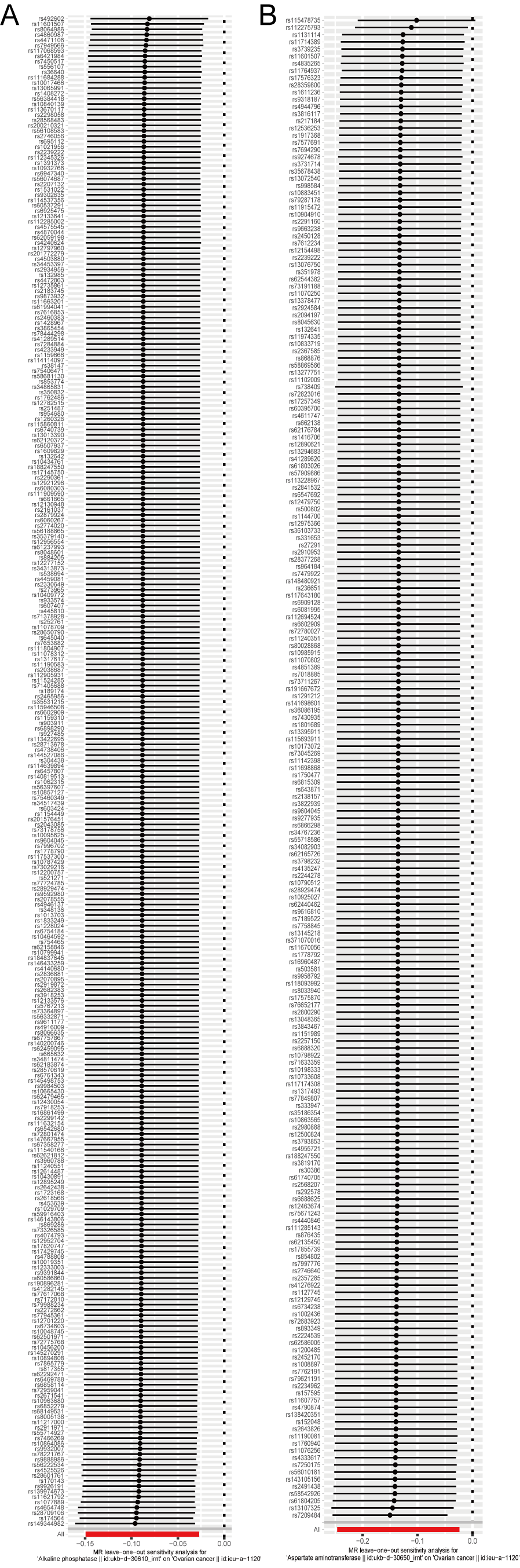

Supplement: Supplementary file 1 — Additional file 1. [file 40246_2024_642_MOESM1_ESM.tif]
