# Supplementary material for: Elucidating the role of liver enzymes as markers and regulators in ovarian cancer: a synergistic approach using Mendelian randomization, single-cell analysis, and clinical evidence
Source: Hum Genomics. 2024 Jun 24;18:71. doi: 10.1186/s40246-024-00642-4 (PMC11197171; doi:10.1186/s40246-024-00642-4)
Supplement: Supplementary file 2 — Additional file 2. [file 40246_2024_642_MOESM2_ESM.ppt]

## Slide 1
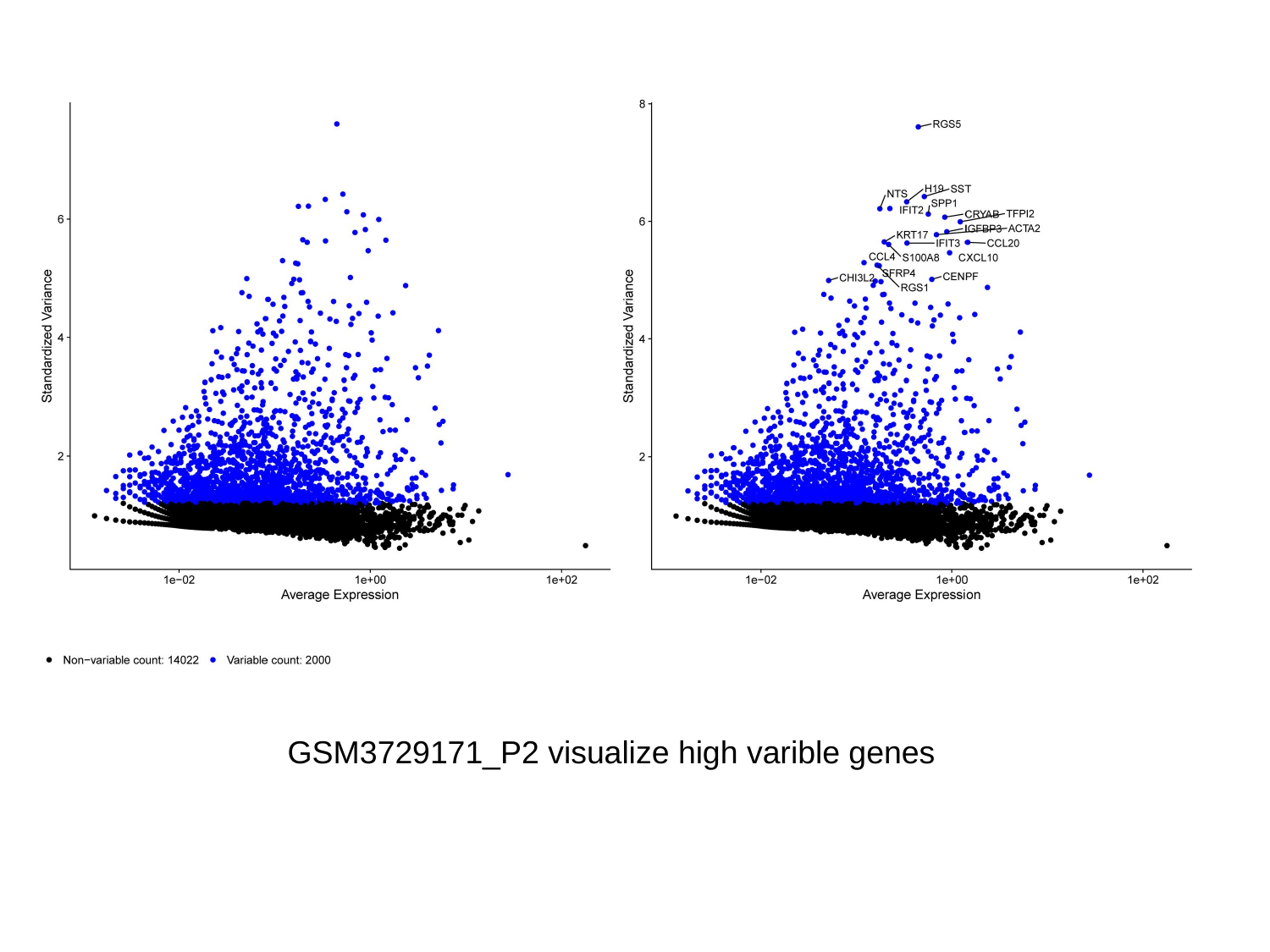

GSM3729171_P2 visualize high varible genes

## Slide 2
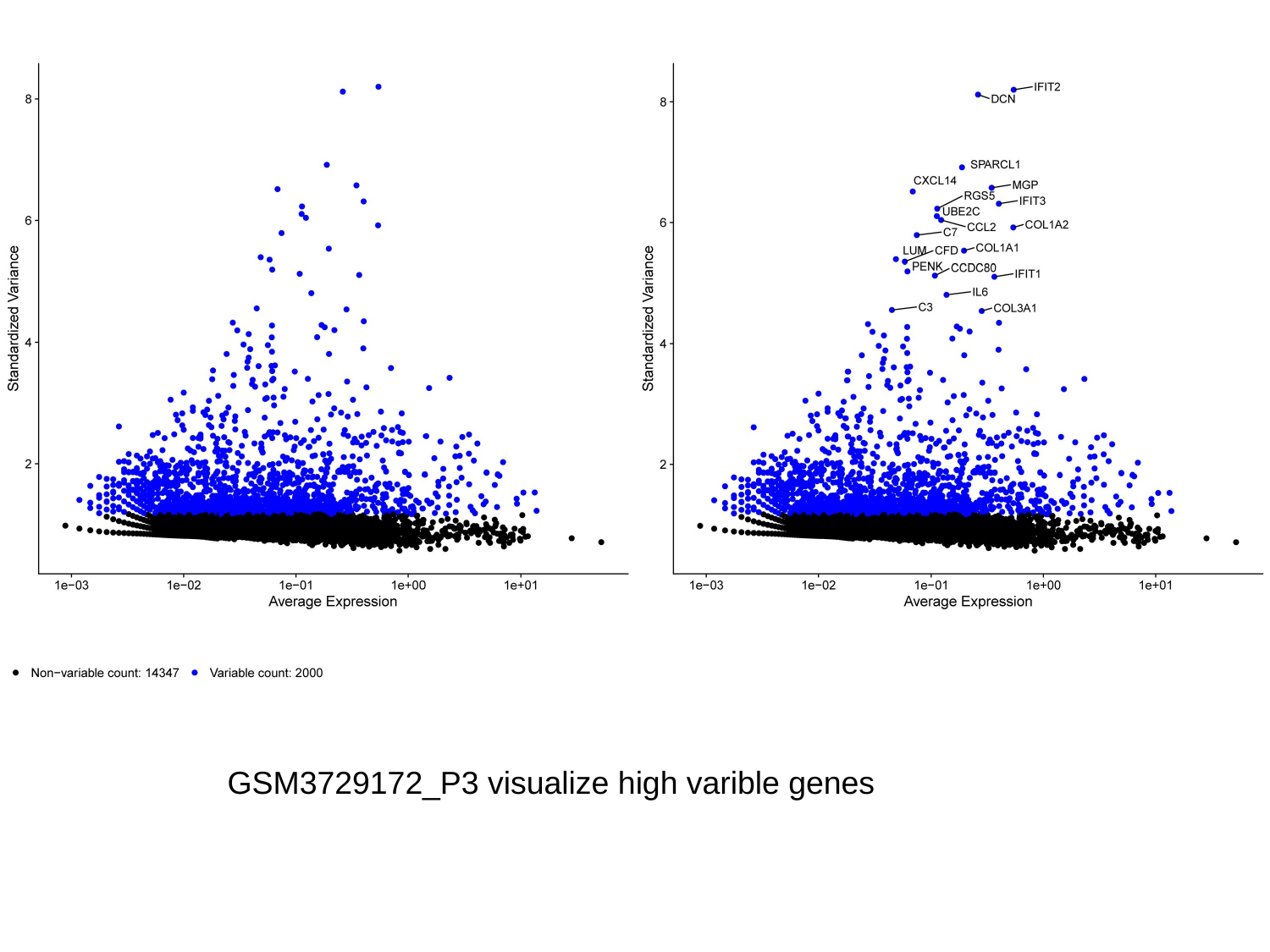

GSM3729172_P3 visualize high varible genes

## Slide 3
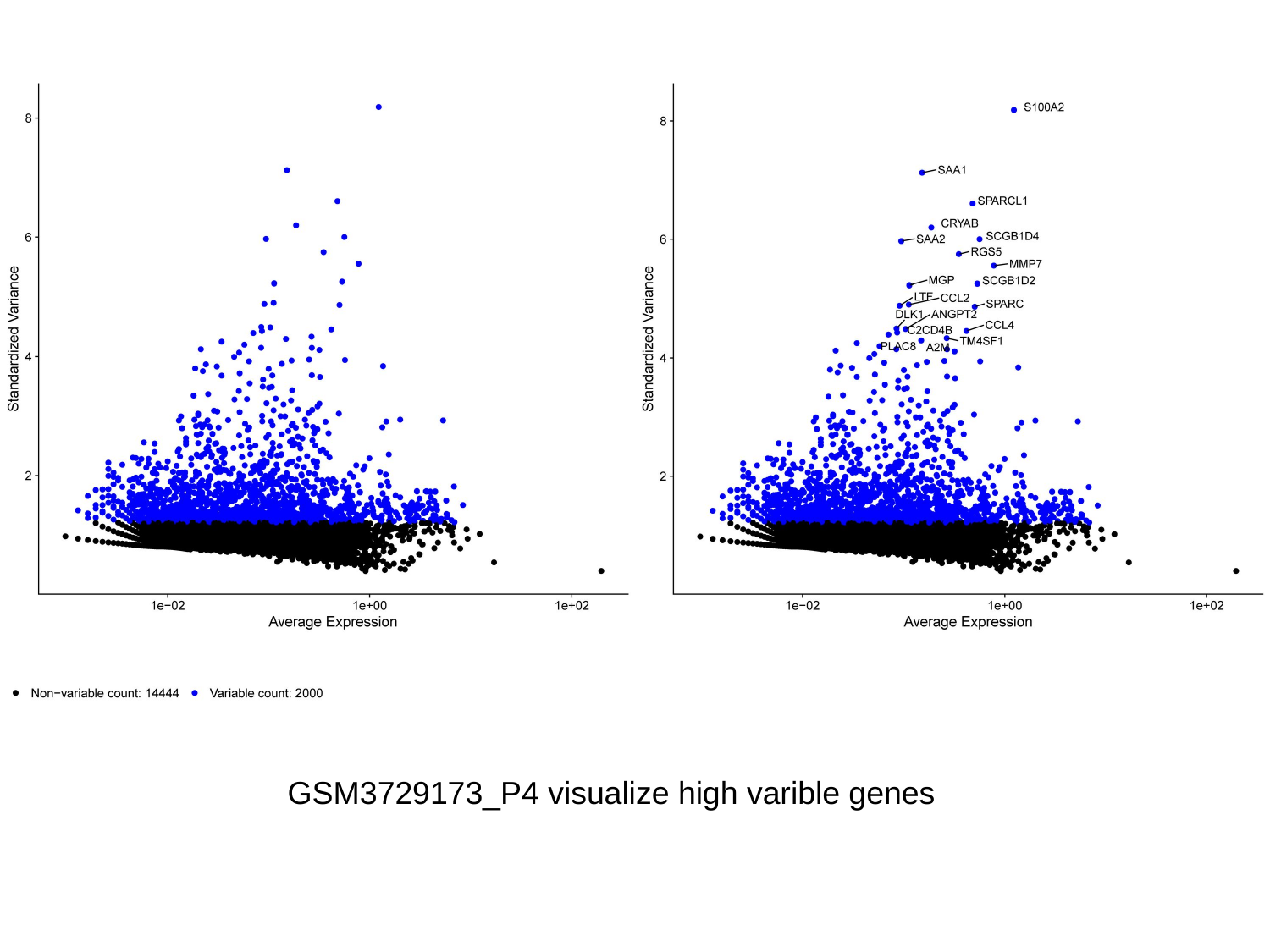

GSM3729173_P4 visualize high varible genes

## Slide 4
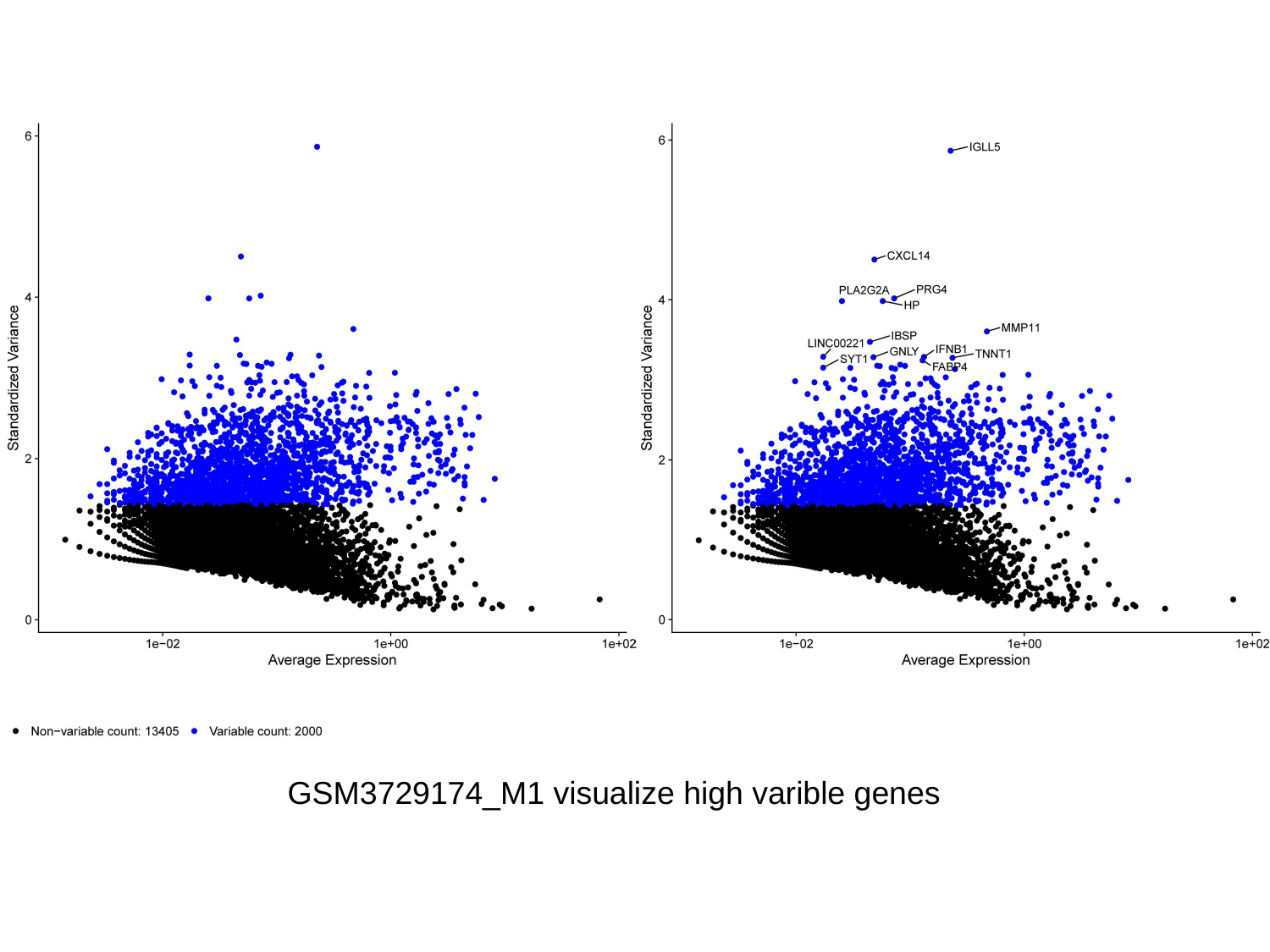

GSM3729174_M1 visualize high varible genes

## Slide 5
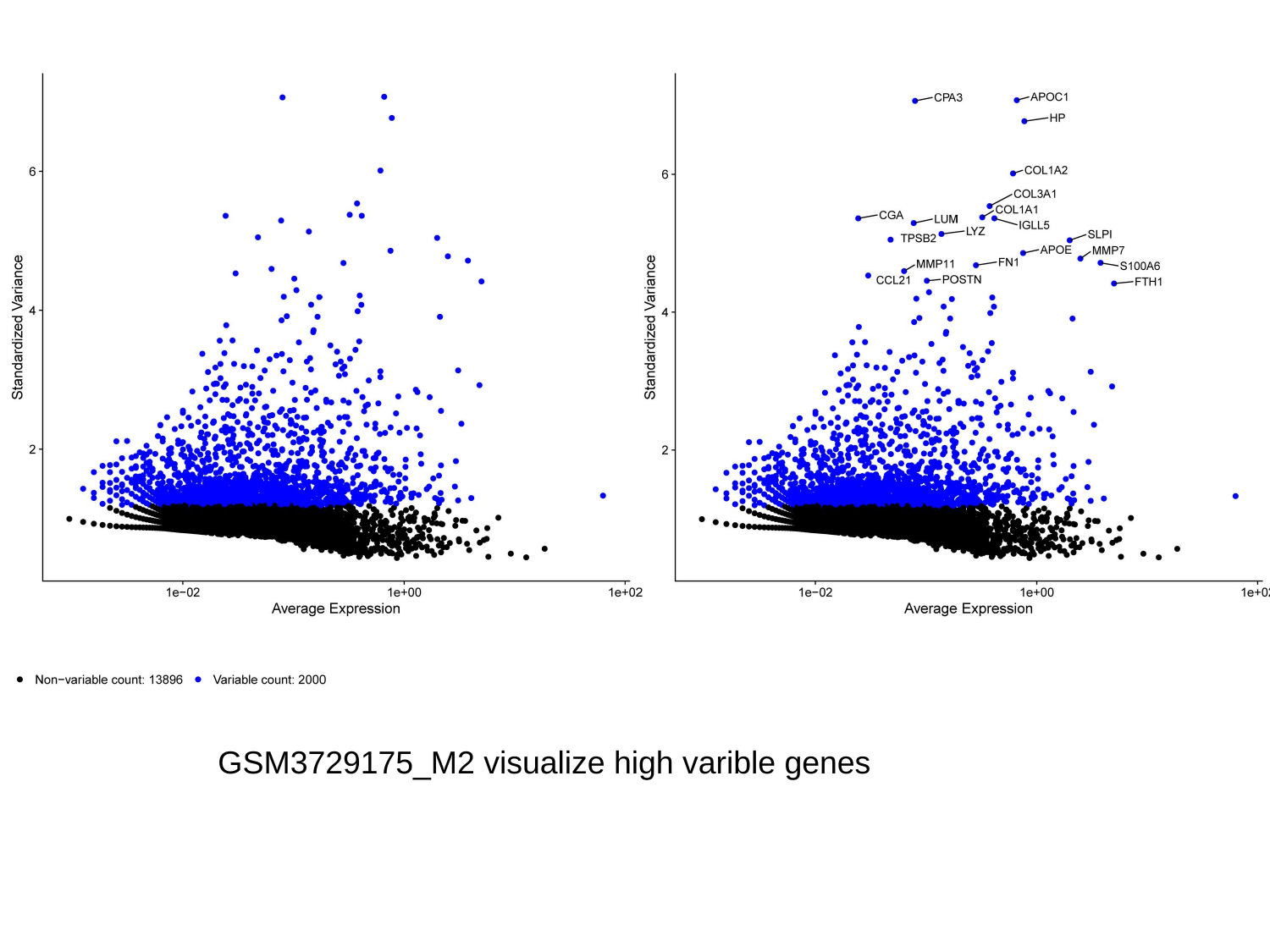

GSM3729175_M2 visualize high varible genes

## Slide 6
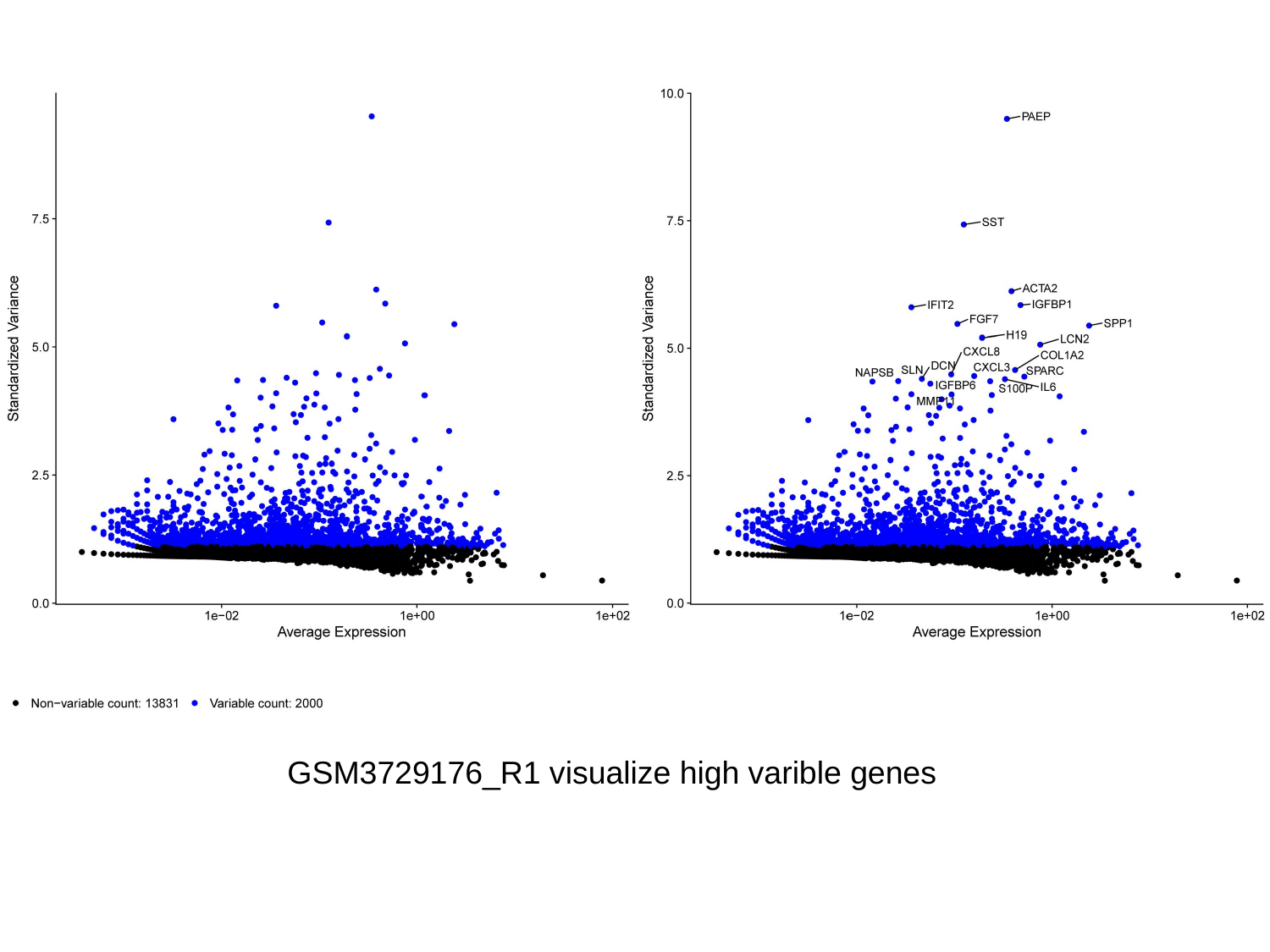

GSM3729176_R1 visualize high varible genes

## Slide 7
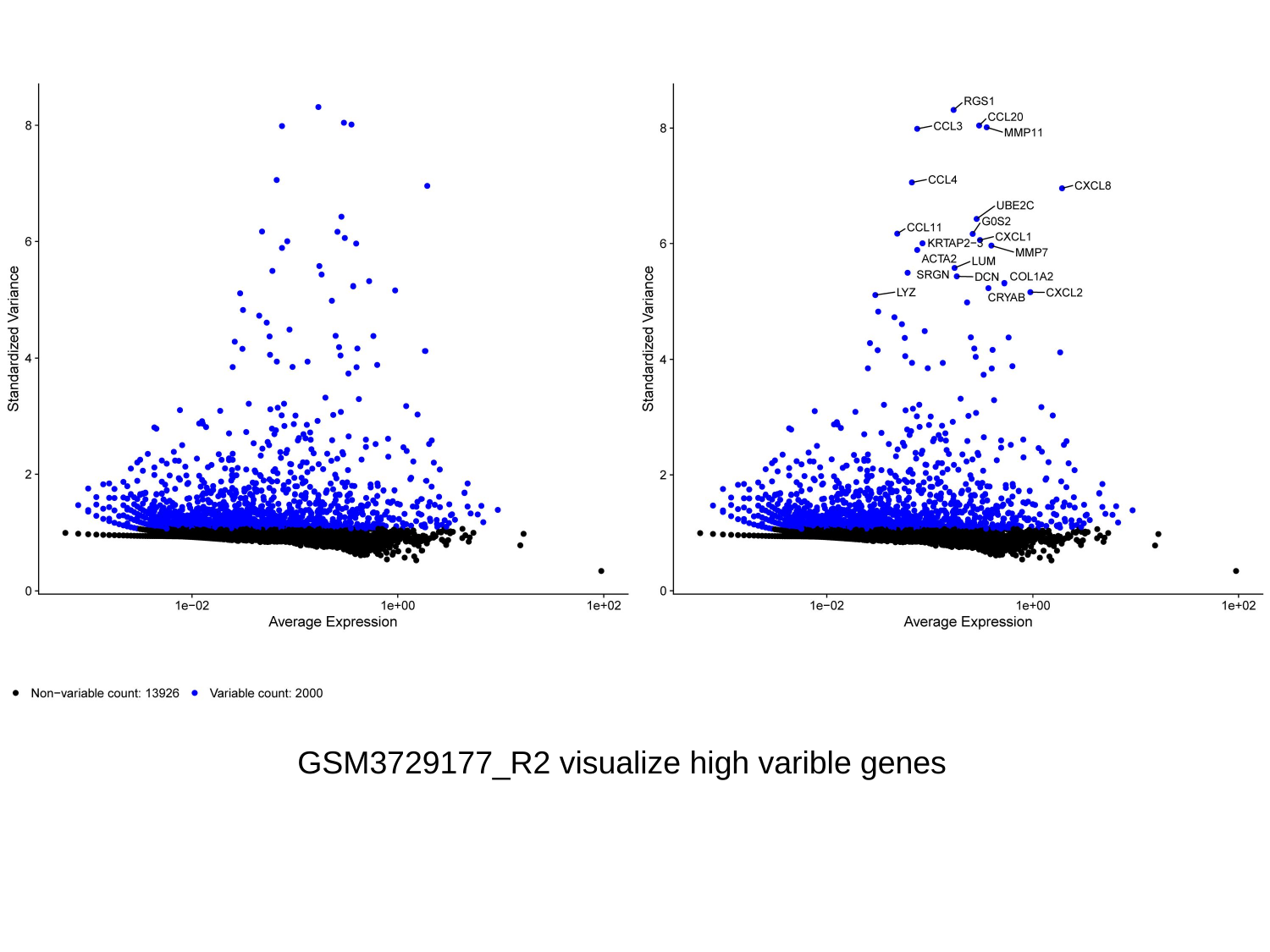

GSM3729177_R2 visualize high varible genes
